# Selenium Deficiency during Pregnancy in Mice Impairs Exercise Performance and Metabolic Function in Adult Offspring

**DOI:** 10.3390/nu14051125

**Published:** 2022-03-07

**Authors:** Pierre Hofstee, Anthony V. Perkins, James S. M. Cuffe

**Affiliations:** 1 School of Medical Science, Menzies Health Institute Queensland, Griffith University, Gold Coast Campus, Southport, QLD 4215, Australia; pierre.hofstee@alumni.griffithuni.edu.au (P.H.); a.perkins@griffith.edu.au (A.V.P.); 2 The School of Biomedical Sciences, The University of Queensland, St. Lucia, QLD 4072, Australia

**Keywords:** physical activity, DOHaD, metabolism, selenoproteins

## Abstract

Selenium deficiency during the perinatal period programs metabolic dysfunction in offspring. Postnatal exercise may prevent the development of programmed metabolic disease. This study investigated the impact of selenium deficiency on offspring exercise behavior and whether this improved metabolic health. Female C57BL/6 mice were randomly allocated to control (NormalSe, >190 μg/Se/kg, *n* = 8) or low-selenium (LowSe, <50 μg/Se/kg, *n* = 8) diets from four weeks before mating. Male offspring were weaned at postnatal day (PN) twenty-four and placed on a normal chow diet. At PN60, mice were placed in cages with bi-directional running wheels and monitored until PN180. LowSe offspring had a reduced average weekly running speed and distance (*p* < 0.05). LowSe offspring exhibited glucose intolerance, with increased peak blood glucose (*p* < 0.05) and area under the curve following an intra-peritoneal injection of glucose (*p* < 0.05). Furthermore, mRNA expression of several selenoproteins within cardiac and skeletal muscle were increased in LowSe offspring (*p* < 0.05). The results indicated that selenium deficiency during development reduces exercise behavior. Furthermore, exercise does not prevent programmed glucose intolerance in low-selenium offspring. This highlights that exercise may not be the optimal intervention for metabolic disease in offspring impacted by selenium deficiency in early life.

## 1. Introduction

Exercise and physical activity are known to stimulate beneficial physiological responses at both the cellular level and across multiple organ systems [1]. Individuals at risk of chronic disease, such as type 2 diabetes mellitus, can benefit significantly from increasing their physical activity, if they are physically capable of doing so. Regular endurance exercise causes adaptations that lead to a shift in oxidative metabolism, upregulating mitochondrial systems, enhancing oxidative capacity and substantially improving mitochondrial respiration [2]. Exercise also facilitates insulin-independent glucose uptake in skeletal muscle so that it can be utilized in cellular respiratory processes [3]—this is known to occur even in individuals who are insulin-resistant and helps to restore insulin sensitivity. Increased energy utilization by tissues, such as cardiac and skeletal muscle during physical activity, is strongly impacted by several essential micronutrients [4] that help maintain optimal mitochondrial respiration and prevent excessive oxidative stress [5]. One micronutrient particularly important to endogenous antioxidant systems is selenium [6].

Selenium is an essential component of ~25 gene products known as selenoproteins, which are necessary for a variety of developmental and metabolic processes, including muscle development and combating oxidative stress [7]. The importance of selenium and selenoproteins in muscle function is well-known, with selenium deficiency having severe ramifications for muscle development, function and remodeling [8]. For example, Keshan disease is a dilated cardiomyopathy associated with severe selenium deficiency [9]. Additionally, selenium deficiency is associated with nutritional muscular dystrophy, characterized by myalgia, muscle weakness and fatigue [10]. Lack of specific selenoproteins is associated with multiple muscle and heart defects. Specifically, SelenoN is linked to genetic disorders inducing congenital muscular dystrophy [11], and SelenoS deficiency is known to impair exercise performance [12].

Recently, the importance of selenium for skeletal muscle development in relation to the Developmental Origins of Health and Disease (DOHaD) has gained interest [13]. We have previously demonstrated that selenium deficiency in pregnancy causes growth restriction of the fetus, programs metabolic disease in offspring and causes long-term changes to selenoprotein expression [14,15]. Male offspring, from mothers that were fed a selenium-deficient diet, developed glucose intolerance and alterations to the insulin signaling pathway in skeletal muscle [14]. This occurred even though the male offspring had received a selenium-replete diet for all their adult life and were only exposed to selenium deficiency due to low maternal selenium intake [14]. Other studies have demonstrated that postnatal exercise can prevent programmed metabolic disease in offspring in animal models of intrauterine growth restriction [16,17]. However, given that selenoprotein deficiencies during adulthood can impair exercise performance, it is important to know if selenium deficiency during fetal development similarly impairs exercise performance in adult offspring. This in turn may limit any benefits that exercise may have in preventing programmed metabolic disease. This study therefore aimed to assess if male offspring from selenium-deficient mothers had any impairments in exercise behavior. Furthermore, we aimed to investigate if postnatal exercise could mitigate the previously reported programmed metabolic dysfunction seen in sedentary offspring of selenium-deficient mothers.

## 2. Materials and Methods

### 2.1. Animal Procedures

Animal procedures, including the composition of the diet used, have been described previously [18]. Animal husbandry and all procedures were conducted in accordance to the DOHaD research “Animals in Research: Reporting In Vivo Experiments” (ARRIVE) guidelines.

Briefly, female C57BL/6 mice were purchased from the Animal Resources Centre (ARC, Perth, Western Australia) and housed in an environmentally controlled environment (23 °C, standard 12 h light/dark cycles). Additional environmental enrichment was provided. After acclimatization for one week, mice were randomly allocated to either a control (NormalSe, >190 µg/Se/kg, *n* = 8) or low-selenium (LowSe, <50 µg/Se/kg, *n* = 8) custom-made diet four weeks prior to mating, throughout gestation and lactation. Complete details of this custom diet have been described previously [18]. To confirm that this dietary manipulation resulted in lower tissue selenium concentrations, we previously measured the liver selenium content in a subset of animals during late pregnancy (NormalSe liver Se content 0.25 µg/g, LowSe liver Se content 0.09 µg/g) [18]. After mice gave birth, offspring were monitored and weighed daily from postnatal (PN) day 8. Offspring were weaned at postnatal day (PN) 24 and placed on a normal animal chow diet (230 µg selenium/kg, Teklad Global 18% Protein Rodent Diet Irradiated, ENVIGO, Madison, WI, USA) and group housed according to sex. All mice had ad libitum access to water and their respective diets.

At PN60, male offspring from NormalSe (*n* = 6) and LowSe (*n* = 6) dams were randomly allocated individually to exercising cages with access to a bi-directional wheel (20 cm-diameter/6.5 cm-wide solid-surface Wodent Wheels, Transoniq, Flagstaff, AZ), allowing voluntary running. Due to a more overt metabolic phenotype present in the male offspring and limited resources, only male mice were assessed in the current study. Additionally, for many parameters, the results for sedentary animals have been previously published [18], and thus no direct comparisons to those animals are made in the current study. The animals used in this study have not been used for any other study.

Running activity and behavior of the exercised group was monitored daily and recorded from calibrated bicycle odometers that detected bi-directional movement via a reed-switch mechanism (model BC-506/509; Sigma Sport, Olney, IL, USA). After 4 months of exercise, at PN180, mice were culled via cervical dislocation. Blood samples were immediately collected by cardiac puncture. Blood was placed into lithium heparin tubes, which were subsequently centrifuged at 2000× *g* for 5 min, and plasma was collected. Tissue was then removed, weighed and snap-frozen in liquid nitrogen and stored at −80 °C.

Glucose levels were measured using an Accu-Chek Performa II Glucometer (Mannheim, Germany) as recently described [14]. At PN90, mice were fasted overnight. The following morning, a tail snip was performed, and fasting blood glucose was measured. At PN170, mice were again fasted overnight, and a tail snip was performed to assess fasting blood glucose prior to an intraperitoneal glucose tolerance test (IGTT). Mice were subsequently administered with 1 g/kg of glucose (D-(+)-Glucose, Sigma-Aldrich, Darmstadt, Germany) in saline (0.9% sodium chloride solution, Sigma-Aldrich) via intraperitoneal (IP) injection (20% Glu in 0.9% NaCl solution). Glucose levels were measured at 0, 30, 60, 90, 120 and 180 min post-injection. Glucose levels were further measured in whole blood at time of death at PN180.

### 2.2. Mitochondrial Respiration

To investigate whether altered exercise capacity was due to impaired mitochondrial function within the heart, mitochondrial respiratory capacity was measured in cardiac apex tissue using the Oxygraph 2k (Oroboros Instruments, Innsbruck, Austria) instrument, as previously described [18]. Briefly, approximately 4 mg of heart tissue was homogenized in mitochondrial respiration media (MiR06—catalase with MiR05—0.5 mM EDTA, 3 mM MgCl2·6H2O, 60 mM K-lactobionate, 20 mM taurine, 10 mM KH2PO4, 20 mM HEPES, 110 mM sucrose, 1 g/L BSA essentially fatty acid free, pH 7.1) in a 5 mL glass dounce (Duall 22, Kimble Chase, Atlanta, GA, USA), and then diluted in MiR06 to 1 mg/mL [19]. Oxygraph 2K chambers were primed and calibrated with MiR06 at 37 °C. After the addition of cardiac tissue homogenate into Oxygraph 2K chambers, mitochondrial respiration was determined using the substrate-uncoupler-inhibitor-titration (SUIT) protocol 1. Electron transport chain (ETC) activity was evaluated using the addition of ETC substrates and inhibitors at specific respiratory states. Pyruvate (5 mM), glutamate (10 mM) and malate (2 mM) were added to determine complex I (CI)-mediated LEAK (non-phosphorylating respiration) respiration. Subsequently, oxidative phosphorylation (OXPHOS) through CI was stimulated by the addition of ADP (1–5 mM). The integrity of the outer mitochondrial membrane was tested with the addition of cytochrome C (10 μM). OXPHOS was then stimulated through complex II (CII) with the addition of succinate (10 mM), which was followed by the uncoupler carbonyl cyanide m-chloro phenyl hydrazone (CCCP, 1 mM). This enabled the determination of ETC capacity with CII-mediated flux, independently. CI was inhibited by the addition of Rotenone (1 μM). Complex III (CIII) was then inhibited by a further addition of antimycin A, allowing for the measurement of residual oxygen consumption (indicating non-ETS respiration). N,N,N′,N′-tetramethyl-p-phenylenediamine dihydrochloride (TMPD, 0.5 mM) and ascorbate (2 mM) were added to determine OXPHOS through complex IV. The obtained quantitative data were indicative of the mitochondrial respiration functional ability of the tissue samples. All data were analyzed using DatLab software (Oroboros Instruments GmbH, Innsbruck, Austria), with respiratory states expressed relative to tissue weight.

### 2.3. Quantitative PCR

The expression of mRNA was determined in both cardiac and skeletal muscle as previously described [14,15]. The RNAeasy mini kit (Qiagen, Melbourne, Australia; Cat. No. 74106), proteinase K (20 mg/mL) and incubation at 55 °C were used to extract RNA. NanoDrop 2000/2000c was used to measure final RNA concentrations. The conversion of RNA into cDNA for qPCR analysis was completed using the Bio-Rad iScript gDNA clear cDNA synthesis kit (Hercules, California, USA; Cat. No. 172-5035). All PCR’s were performed with thermocycling parameters as follows: initial activation step at 95 °C for 2 min, followed by 40 cycles of denaturation at 95 °C for 5 s and combined annealing/extension at 60 °C for 10 s on the StepOne real-time PCR system (Applied Biosystems). All samples were run in duplicate, with qPCR performed using 20 ng of cDNA in 10 µL reactions. All PCR reactions were performed in correspondence with the MIQE guidelines [20]. KiCqStart SYBR green PCR primers (Sigma-Aldrich, St. Louis, MO, USA, Table 1) were used to measure the mRNA expression of selenium-dependent genes. No product was detected in non-template controls and melt curve analysis demonstrated single spikes for all genes. To ensure accuracy, several potential reference genes were utilized for assessment, with the two genes selected as housekeepers demonstrating consistent expression in skeletal muscle without any impact by treatment. All expression data were normalized to the geometric mean of the reference genes: hypoxanthine phosphoribosyltransferase 1 (Hprt1, NM_013556) and beta-2-microglobulin (B2m, NM_009735). The final expression was calculated using the 2-ΔΔCt method, with *n* = 6 per group.

### 2.4. Statistical Analysis

All data at PN180 were checked for normality and differences in variance before undergoing an unpaired *t*-test if normally distributed, or a Mann–Whitney test if not normally distributed (GraphPad Prism 9.2; RRID:SCR 002798), comparing LowSe and NormalSe offspring from the exercised cohort. Cumulative running distance was analyzed by a two-way repeated measures analysis of variance (ANOVA). The main effects analyzed were maternal selenium status (Treatment, Ptrt) and time (Time, Ptime), with any interactions between treatment and time also assessed (Interaction, Pint). When a major effect of treatment, time or an interaction between treatment and time was detected, Sidak post hoc analysis was performed. All data are presented as means ± SEM and *p* < 0.05 was considered statistically significant.

## 3. Results

### 3.1. Offspring Weight, Food and Water Consumption

**Figure 1:**
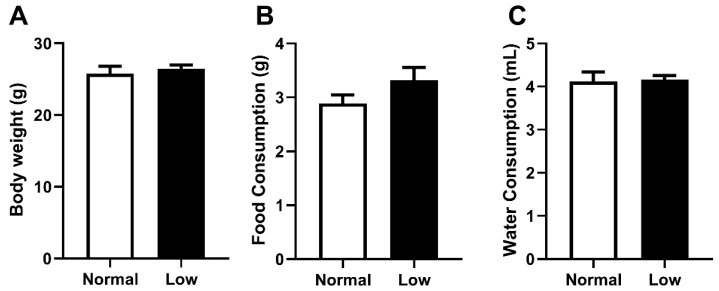
Exercising mice body weight, food and water consumption. Exercising male mice (**A**) body weight at PN180 as well as average (**B**) food and (**C**) water consumption from weaning to PN180. Data are mean ± SEM and analyzed by unpaired *t*-test. Significance determined by *p* < 0.05. n = 6.

As selenium deficiency may have impacted offspring body weight and dietary behavior differently in an exercising cohort compared to our previous sedentary cohort, these parameters were carefully monitored [14]. Body weight at PN180 (Figure 1A), as well as food (Figure 1B) and water (Figure 1C) consumption from weaning until PN180, were not different between treatment groups. At PN180, there were no differences in weight of any organs between animals from the NormalSe and LowSe exercising groups (Table 2).

### 3.2. Exercise Behavior

**Figure 2:**
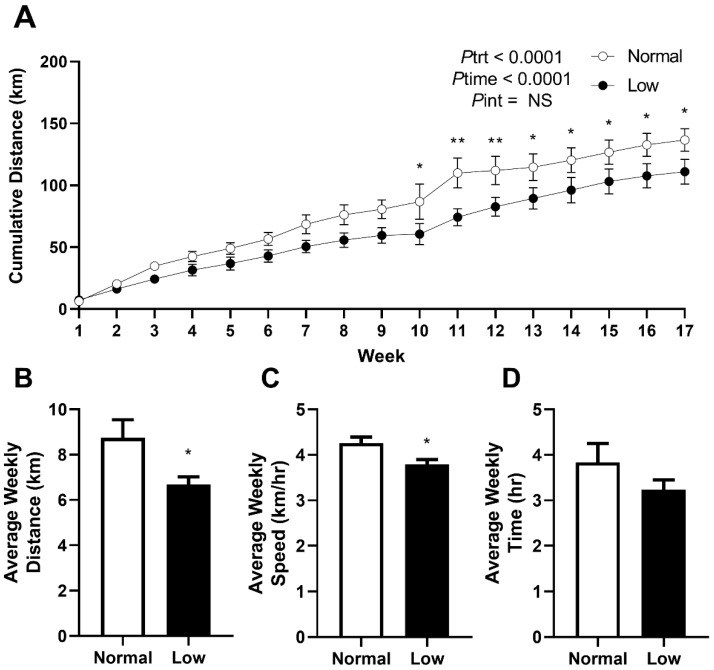
Exercise behavior from PN60 to PN180. Exercise behavior in male mice from normal and low-selenium litters. (**A**) Cumulative running distance from PN60 to PN180. (**B**) Average weekly running distance, (**C**) speed and (**D**) time over four months. Cumulative data are mean ± SEM and analyzed by two-way ANOVA with treatment (Ptrt) and time (Ptime) as major factors. Pint represents the interaction between Ptrt and Ptime. Multiple comparisons were determined by Sidak post hoc testing. Distance, speed and time data were analyzed by unpaired *t*-test. * = *p* < 0.05, ** = *p* < 0.01 compared to the control exercise group. n= 6.

Mice were allocated to cages with 24 h access to a bi-directional running wheel from PN60 to monitor voluntary exercise behavior and to determine if exercise would prevent the programmed outcomes previously reported in offspring of selenium-deficient mothers. Cumulative voluntary running distance (Figure 2A) was significantly lower (Ptrt < 0.0001) in the LowSe group compared to the NormalSe group. Post hoc analysis demonstrated that this reduction in cumulative distance began approximately from 10 weeks of exercise (*p* < 0.05). LowSe offspring also had a reduced (*p* < 0.05) average weekly running distance (Figure 2B) and speed (Figure 2C) over the four-month period. There was no difference in the average time (Figure 2D) spent running each week between the two groups.

### 3.3. Glucose Metabolism

**Figure 3:**
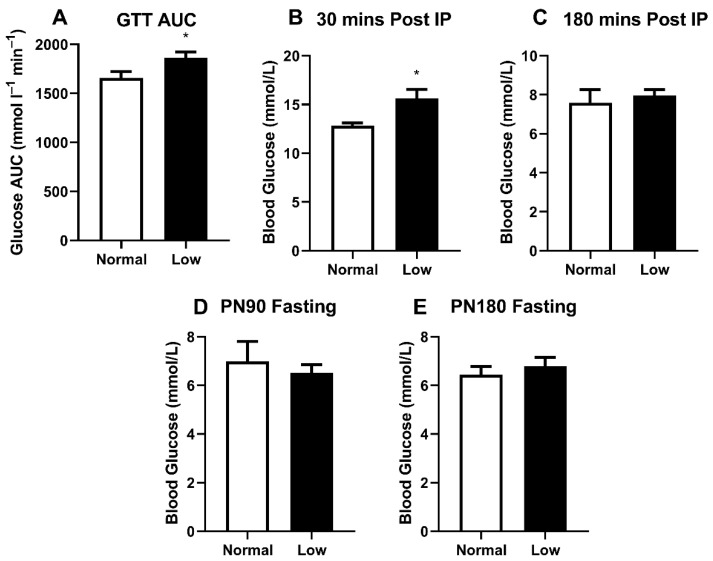
Glucose tolerance and resting blood glucose analysis. PN170 glucose tolerance testing showing (**A**) GTT AUC as well as (**B**) peak blood glucose 30 min post-IP and (**C**) 180 min post-IP. (**D**) PN90 and (**E**) PN180 fasting blood glucose levels. Data are mean ± SEM and analyzed by unpaired *t*-test. * = *p* < 0.05. *n* = 6 per group.

We previously demonstrated glucose intolerance in sedentary LowSe offspring [14], but anticipated that this may be modified in exercising animals. LowSe offspring provided with running wheels had a greater area under the glucose tolerance curve (Figure 3A) (*p* < 0.05) compared to exercising NormalSe offspring. Furthermore, approximately 30 min post-IP glucose injection, peak blood glucose (Figure 3B) was also greater (*p* < 0.05) in LowSe offspring compared to NormalSe offspring. Three hours post-IP injection of glucose, blood glucose levels were no longer different between LowSe and NormalSe offspring (Figure 3C). Fasting blood glucose levels were also determined at PN90 (Figure 3D) and PN180 (Figure 3E); however, these were not different between groups.

### 3.4. Selenoprotein Expression in the Heart and Gastrocnemii

One mechanism by which early-life selenium deficiency may impair exercise performance and disturb metabolic processes is by causing long-term changes to the expression of selenoproteins. Given the important roles of both heart and muscle tissue in exercise performance, we assessed the mRNA expression of selenoproteins in heart and gastrocnemius tissue. For ease of interpretation, data are represented as arbitrary units, and thus comparisons are only applicable between the same selenoprotein in a single tissue (Table 3). The mRNA expressions of SelenoF, SelenoS and SelenoP were increased (*p* < 0.05) in hearts of exercising mice from LowSe litters compared to NormalSe litters. SelenoF mRNA expression was higher (*p* < 0.05) in the gastrocnemius of the LowSe offspring compared to offspring from the NormalSe group.

### 3.5. Mitochondrial Respiration of the Heart

Given that a greater number of selenoproteins were impacted in cardiac tissue compared to muscle tissue, we assessed mitochondrial respiratory capacity in the heart. Although the oxidative phosphorylation capacity of complex I, complex II and electron transport capacity at PN180 appeared to be decreased in mitochondria from the apex of the heart of exercising mice from selenium-deficient litters, this did not reach significance (Table 4). Additionally, there were no changes in flux control factors at PN180.

## 4. Discussion

The effect of exercise on attenuating metabolic disease is well-documented [21]; however, exercise-induced improvements in health may be limited by programmed impairments in exercise capacity. Selenium deficiency during adult life has previously been associated with reduced muscle function [8] although the impact of early-life selenium deficiency on later-life exercise capacity was unknown before this study. We previously demonstrated that selenium deficiency in utero caused aberrant metabolic processes in skeletal muscle of offspring and atypical selenoprotein expression in several tissues [14,15,18]. In the current study, we demonstrated that selenium deficiency during pregnancy and up until weaning resulted in a reduced running distance and running speed throughout the adult life. This occurred despite mice consuming a selenium-replete diet from weaning onwards. We also demonstrated that the diabetic-like phenotype seen previously in sedentary LowSe offspring was also present in exercising LowSe offspring. This phenotype appears to be unrelated to changes in cardiac respiratory function. Furthermore, long-term changes to selenoprotein mRNA levels appear to be compensatory processes that do not directly contribute to either the glucose intolerance or the impaired exercise performance.

This study clearly demonstrated that LowSe offspring have a reduced running speed and running distance on the voluntary running wheel. While we interpreted this as impaired exercise capacity, it is possible that it may reflect a different phenotype. Mice are said to enjoy wheel running, and it is possible that changes to neurological circuits related to reward may alter the running time of mice. Similarly, behaviors relating to stress and depression may impact the running distance [22]. The fact that mice from both groups spent a similar amount of time running supports the idea that mice in the current study have reduced exercise performance with reduced exercise output, which would in turn minimize any potential benefits of exercise for improving metabolic health.

Previously, we demonstrated that sedentary male mice from selenium-deficient litters had reduced glucose tolerance and increased insulin concentrations, concomitant with reduced GLUT4 protein expression and a blunting of several genes involved in the insulin receptor pathway in skeletal muscle, including the insulin receptor [14]. Given the fact that LowSe offspring had significantly reduced exercise performance across their adult lifespan in the current study, it is not surprising that they displayed glucose intolerance similar to their sedentary counterparts. Previous studies have demonstrated that animal models of muscle disease, such as the mdx dystrophic mouse, are commonly glucose-intolerant [23], and we know that a sedentary lifestyle contributes to the development of type 2 diabetes mellitus [24]. It is possible that the reduced exercise performance in the LowSe group of the current study may be one of multiple factors contributing to systemic glucose intolerance. While not previously assessed, it is possible that LowSe mice from the previous study also displayed reduced activity within their cages. Physical activity is known to increase glucose uptake in skeletal muscle through insulin-independent translocation of GLUT4 to the cell surface. This adaptation is generally associated with increased insulin sensitivity [3]. Given that our previous findings are suggestive of reduced insulin sensitivity, it is likely that reduced exercise is just one of multiple factors driving impaired glucose control.

Interestingly, mRNA expression of several selenoproteins was elevated in heart and muscle tissue, suggesting that the early-life exposure has permanently impacted selenium regulation in these tissues. Two of the selenoproteins upregulated in cardiac tissue, SelenoF and SelenoS, reside in the endoplasmic reticulum. There is significant evidence supporting the antioxidant function of SelenoS, with overexpression associated with protection against hydrogen peroxide-induced cell toxicity [25]. Interestingly, mice with global SelenoS deletion have been shown to have impaired exercise capacity, with the mechanism involved not completely understood [12]. It is possible that SelenoS protein levels in the hearts of mice from the current study have been depleted by early-life selenium deficiency, and that the increased SelenoS mRNA is a compensatory change attempting to restore SelenoS protein concentrations. Such a change may help explain the impaired exercise capacity in the current model; however, this would require significant further investigation. Interestingly, we have previously demonstrated that although SelenoS mRNA expression is reduced in the maternal livers of mice during the period of selenium deficiency [15], its expression is elevated in the fetal heart at the end of pregnancy. This suggests that any deficits in SelenoS protein levels may have occurred during the early stages of heart development. SelenoF mRNA levels were also increased in the heart and muscle of LowSe offspring. SelenoF is involved in glycoprotein folding and increases during protective cellular functions that relate to endoplasmic reticulum stress [26]. It is likely that the increased mRNA expression of this selenoprotein may be an adaptive response to cellular stress. SelenoP expression was also increased in the heart, and elevations in this selenoprotein are known to be associated with improved distribution of selenium to needed tissues [27]. The increase in SelenoP may be an adaptive response to bring in selenium to restore concentrations in cardiac tissue.

When we investigated mitochondrial respiration in heart tissue from exercised mice, no differences were identified. Previous studies have demonstrated that administration of selenium improves mitochondrial respiration in a range of cell types, including heart cells [28,29]. It is possible that the increased mRNA expression of selenoproteins is a compensatory response to early-life selenium deficiency and is providing some degree of protection against mitochondrial dysfunction in the heart.

Several of the selenoproteins measured by qPCR in the current study are important regulators of oxidative stress. We have previously shown that mRNA expression of key antioxidant selenoproteins (Gpx1, Gpx3, Trxr1 and Trxr2) was decreased in maternal tissues during the period of selenium deficiency [15], but that six-month-old offspring of selenium-deficient dams had similar expression levels compared to offspring from normal selenium mothers. We did however previously demonstrate that Gpx3 mRNA expression was lower in heart tissue of six-month-old sedentary offspring, with the main effect being present in females but not males. In the current study, the mRNA expression of all selenoprotein antioxidants in hearts of six-month-old male exercising offspring was similar between control and selenium-deficient groups, reflecting the data from our previous publication. Given the importance of antioxidants for maintaining cellular function, it is likely that the postnatal provision of dietary selenium has largely restored any early deficits in the expression of these selenoproteins, therefore protecting cardiac tissue from oxidative stress. However, we did not measure any markers of oxidative stress which may have confirmed this hypothesis.

We have previously reported that both glucose intolerance and other disease parameters such as renal dysfunction may be occurring in this model due to impaired thyroid function [14,30]. Similarly, the reduced exercise performance in the current model may relate to impaired thyroid function. Indeed, there is a reciprocal relationship between exercise and thyroid hormone levels, with low thyroid hormone causing impaired exercise performance [31], while voluntary exercise in rats is known to stimulate thyroid hormone production [32]. The fact that no overt differences in exercise occurred in our sedentary model suggests that the low thyroid hormone may precede the impaired exercise performance, rather than low exercise contributing to the low thyroid hormone. Given that the thyroid hormone was not assessed in the exercised cohort, this remains unknown.

Unfortunately, this study was only able to be performed in male mice. We previously identified slight sex differences in disease progression in this model, and it would be of interest to characterize whether female offspring also displayed impaired exercise performance. It would also be of interest to compare the results of voluntary running undertaken in this study to the effects of forced exercise, which may have a greater impact of improving metabolic outcomes. Future studies may also characterize the long-term impact of prenatal selenium deficiency on respiratory capacity in offspring skeletal muscle, as such defects may also contribute to impaired exercise capacity.

## 5. Conclusions

This study demonstrated that mice prenatally exposed to low levels of selenium are either incapable or unwilling to undertake the same degree of exercise compared to mice provided a standard diet. This highlights that while proposing exercise as an intervention for programmed metabolic disease is good in principle, there may be programmed biological reasons why exercise is unable to prevent programmed metabolic disease. Given the high prevalence of selenium deficiency around the world, it is possible that many individuals have been exposed to selenium deficiency in utero. It is unknown if the long-term impact of selenium deficiency on exercise similarly occurs in humans. This data adds to the growing body of evidence highlighting the need to obtain sufficient micronutrients during pregnancy and early postnatal life.

## Figures and Tables

**Table 1:** qPCR primer list.

Group	Gene Name	Gene Symbol	Accession Number	Primer Sequence
Selenoproteins	Thioredoxin Reductase 1	*Txnrd1*	NM_001042513	F’ TCCCAACGAAAATTGAACAG R’ TGTTAAATTCGCCCTCTATG
	Thioredoxin Reductase 2	*Txnrd2*	NM_013711	F’ GAATCACAAGTGACGACATC R’ AAAGATGACATTTGCTGGTC
	Glutathione Peroxidase 1	*Gpx1*	NM_008160	F’ GGAGAATGGCAAGAATGAAG R’ TTCGCACTTCTCAAACAATG
	Glutathione Peroxidase 3	*Gpx3*	NM_008161	F’ ACAAGAGAAGTCTAAGACAGAC R’ TGTAGTGCATTCAGTTCAAG
	Iodothyronine Deiodinase Type 2	*Dio2*	NM_010050	F’ CAGTCTTTTTCTCCAACTGC R’ CCAGTTTAACCTGTTTGTAGG
	Selenoprotein F	*SelenoF*	NM_053102	F’ CTACAGATCAAGTATGTTCGAG R’ TATATGCGTTCCAACTTCTC
	Selenoprotein S	*SelenoS*	NM_024439	F’ ACCTGATGTTGTTGTTAGC R’ CTCTTCTTCAAGCTGTCTTAG
	Selenoprotein K	*SelenoK*	NM_019979	F’ TGATTCCAGATACGACGATG R’ CATTTACCTTCCTCATCCAC
	Selenoprotein M	*SelenoM*	NM_053267	F’ GACAGTTGAATCGCCTAAAG R’ TGGTAATTTCGGCTTAACAG
	Selenoprotein T	*SelenoT*	NM_001040396	F’ GTTCCAGATTTGTGTATCCTG R’ GTGTCTATAAATTGGTTGAGGG
	Selenoprotein N	*SelenoN*	NM_029100	F’ CTTCAAGAAGGTCAACTACC R’ AGCAAGATGGAATGAACAAG
	Selenoprotein P	*SelenoP*	NM_001042613	F’ ATGACTTCCTCATCTATGACAG R’ GAGGTCACAGTTTACAGAAG
House Keepers	Beta-Actin	*Actb*	NM_007393	F’ GATGTATGAAGGCTTTGGTC R’ TGTGCACTTTTATTGGTCTC
	Beta-2-Microglobulin	*B2m*	NM_009735	F’ GTATGCTATCCAGAAAACCC R’ CTGAAGGACATATCTGACATC
	Hypoxanthine Phosphoribosyltransferase 1	*Hprt1*	NM_013556	F’ AGGGATTTGAATCACGTTTG R’ TTTACTGGCAACATCAACAG

**Table 2:** PN180 organ weights of exercising male offspring from mothers on a normal selenium compared to a low-selenium diet.

	Normal	Low	*p*
Heart	149.15 ± 0.10	186.90 ± 19.4	NS
Liver	1308.54 ± 48.91	1322.95 ± 98.30	NS
Kidney	160.01 ± 7.11	171.63 ± 4.10	NS
Adrenal	1.64 ± 0.10	2.07 ± 0.21	NS
Brain	419.85 ± 10.46	438.82 ± 7.96	NS
Gastrocnemius	133.06 ± 3.83	149.05 ± 8.98	NS
Tibialis Anterior	42.37 ± 4.00	44.98 ± 0.92	NS
Soleus	9.33 ± 0.73	10.17 ± 0.32	NS
EDL	9.90 ± 0.32	9.31 ± 0.66	NS
Testes	88.97 ± 2.23	87.30 ± 3.05	NS

Data are mean ± SEM with all weights in milligrams. All measurements of kidneys, adrenals, gastrocnemius, tibialis anterior, soleus, extensor digitorum longus and testes include total weight of left and right organs. Data were analyzed by unpaired *t*-test. n = 6. EDL, extensor digitorum longus; NS, not significant.

**Table 3:** Expression of selenoproteins within the heart and gastrocnemius of exercising mice at PN180.

	Heart			Gastrocnemius		
	Normal	Low	*p*	Normal	Low	*p*
*TrxR1*	1.00 ± 0.16	1.20 ± 0.21	NS	1.00 ± 0.43	1.12 ± 0.54	NS
*TrxR2*	1.00 ± 0.13	1.39 ± 0.25	NS	1.00 ± 0.48	0.84 ± 0.46	NS
*GPx1*	1.00 ± 0.21	1.03 ± 0.17	NS	1.00 ± 0.21	1.81 ± 0.62	NS
*GPx3*	1.00 ± 0.34	0.89 ± 0.32	NS	1.00 ± 0.46	1.03 ± 0.32	NS
*DIO2*	1.00 ± 0.13	0.90 ± 0.24	NS	-	-	-
*SelenoF*	1.00 ± 0.24	1.83 ± 0.19	0.047	1.00 ± 0.13	1.66 ± 0.26	0.030
*SelenoS*	1.00 ± 0.19	1.95 ± 0.31	0.028	1.00 ± 0.46	1.05 ± 0.25	NS
*SelenoK*	1.00 ± 0.25	1.09 ± 0.17	NS	1.00 ± 0.27	1.15 ± 0.35	NS
*SelenoM*	1.00 ± 0.27	0.87 ± 0.18	NS	1.00 ± 0.48	1.76 ± 0.82	NS
*SelenoT*	1.00 ± 0.26	0.58 ± 0.09	NS	1.00 ± 0.20	0.99 ± 0.18	NS
*SelenoN*	1.00 ± 0.37	0.93 ± 0.29	NS	-	-	-
*SelenoP*	1.00 ± 0.25	1.82 ± 0.10	0.030	1.00 ± 0.18	1.42 ± 0.20	NS

Data are mean ± SEM arbitrary units analyzed by unpaired *t*-test. Bold text indicates significance (*p* < 0.05). n = 6 per group. - indicates expression was not determined. TrxR, thioredoxin reductase; GPx, glutathione peroxidase; DIO, iodothyronine deiodinase; Sel, selenoprotein; NS, not significant.

**Table 4:** Mitochondrial respiration capacity within the apex of the heart of exercising mice on normal or low-selenium diets.

		Normal	Low	*p*
**Fluxes**	CI	140.61 ± 56.68	88.66 ± 40.43	NS
	Cic	210.22 ± 68.65	173.20 ± 67.00	NS
	CII	523.47 ± 60.75	381.87 ± 108.53	NS
	ETS	517.33 ± 61.40	402.62 ± 120.83	NS
	CIV	832.62 ± 146.89	1017.73 ± 242.13	NS
	ROX	7.18 ± 2.29	7.89 ± 4.78	NS
Flux control factors (FCF)	FCF CI	0.24 ± 0.08	0.20 ± 0.04	NS
	FCF CI + CII	0.78 ± 0.08	0.78 ± 0.06	NS
	FCFc	0.45 ± 0.08	0.41 ± 0.10	NS
	L/ETS	0.04 ± 0.00	0.11 ± 0.04	NS

Data are mean ± SEM and analyzed by unpaired *t*-test. n = 6 per group. CI, complex I-linked respiration; Cic, cytochrome control factor; CII, complex II-linked respiration; ETS, electron transfer capacity; CIV, complex IV-linked respiration; ROX, residual oxygen capacity; NS, not significant. In addition to these parameters collected following the SUIT protocol, flux control factors (FCF) were assessed. FCF CI (flux control factor; OXPHOS CI as a proportion of maximal respiratory capacity), LCR/CI (LEAK control ratio to OXPHOS CI), FCFc (flux control factor of cytochrome c), L/ETS (LEAK control ratio to ETS capacity). NS, not significant.

## Data Availability

All data are available upon request.
